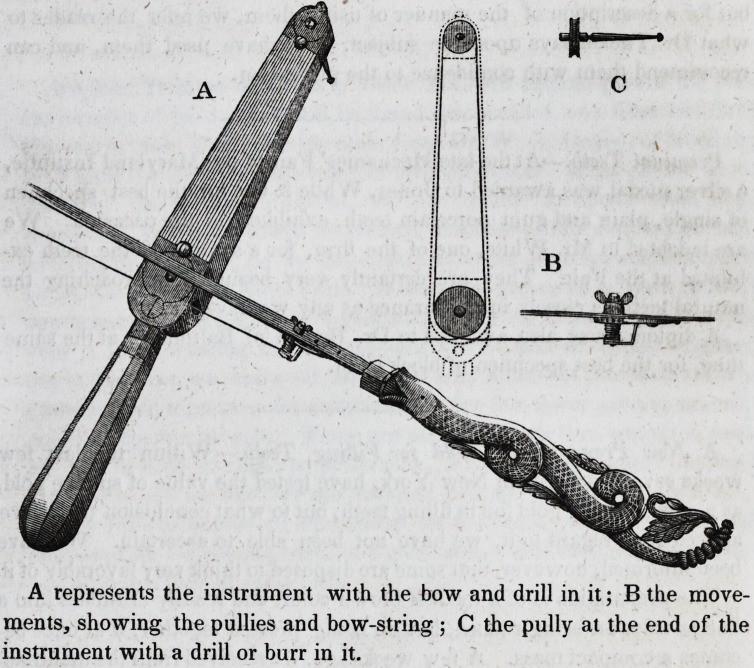# New Drill-Stock

**Published:** 1850-10

**Authors:** 


*New Drill-Stock.*—We are indebted to Dr. W. W. H. Thackston, of Farmville, Va., for a very beautiful drill-stock, which he has recently invented for opening cavities in molar teeth, when the mouth of the patient is too small to be conveniently reached and efficiently acted upon with the instruments ordinarily employed for this purpose. We have tried it in one or two cases, and it worked remarkably well. The instrument was manufactured by Mr. F. Arnold, of Baltimore, and is represented in the cut below.

A represents the instrument with the bow and drill in it; B the movements, showing the pullies and bow-string; C the pully at the end of the instrument with a drill or burr in it.

Although it is seldom that an instrument of this kind is needed, yet, cases do occasionally occur in which one may be advantageously employed. We met with a case, not long since, of a young lady, who, from salivation, had lost, almost wholly, the use of the sphincter muscle of the mouth. The opening was so small as to render it nearly impossible to act efficiently upon her molar teeth, with any other description of instrument, and there are cases, too, where the mouth is naturally so small as to render the use of a straight burr drill exceedingly difficult, if not wholly impossible.

## Figures and Tables

**Figure f1:**